# Cellular pathophysiology of Duchenne muscular dystrophy: insights from a novel rhesus macaque model

**DOI:** 10.1038/s41392-024-02061-2

**Published:** 2024-12-07

**Authors:** Alessandra Moretti, Christian Kupatt, Eckhard Wolf

**Affiliations:** 1 Medical Department I, Cardiology, Angiology, Pneumology, Klinikum rechts der Isar, Technical University of Munich, School of Medicine and Health, 81675 Munich, Germany; 2 DZHK (German Center of Cardiovascular Research), Munich Heart Alliance, 80336 Munich, Germany; 3 Gene Center and Center for Innovative Medical Models (CiMM), LMU Munich, 81377 Munich, Germany; 4 Interfaculty Center for Endocrine and Cardiovascular Disease Network Modelling and Clinical Transfer (ICONLMU), LMU Munich, 81377 Munich, Germany

**Keywords:** Disease model, Translational research

In a recent study published in *Cell*, Ren and colleagues^1^ present a new rhesus macaque (*Macaca mulatta*) model for Duchenne muscular dystrophy (DMD) and a comprehensive single-cell analysis of skeletal muscle, providing detailed insights into the cellular pathophysiology.

DMD is a fatal X-linked disorder caused by loss-of-function mutations in the *DMD* gene, leading to the absence of dystrophin and progressive degeneration of skeletal and cardiac muscle.^2^ Animal models have been essential for studying disease mechanisms and testing targeted therapies, such as exon skipping or gene editing to restore the *DMD* gene’s reading frame and dystrophin production. The *mdx* mouse, which harbors a nonsense mutation in exon 23 of *Dmd*, or genetically engineered mouse models mimicking human *DMD* mutations, are widely used but have limitations for translational studies due to their small size and relatively mild phenotype.^3^ Consequently, several lines of dogs with spontaneous *DMD* mutations and genetically engineered porcine DMD models are also used.^4,5^

**Fig. 1:**
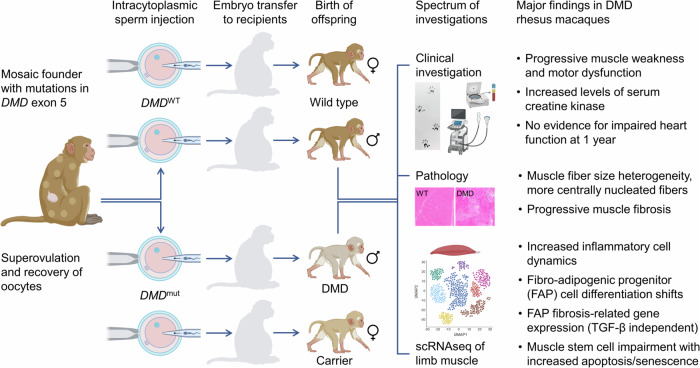
Generation and characterization of a new rhesus macaque model for Duchenne muscular dystrophy (DMD). The figure was created with BioRender.com

Ren and colleagues^1^ make an important addition to the available DMD models (Fig. 1). A total of 6 DMD rhesus macaques were generated by intracytoplasmic sperm injection (ICSI) into oocytes recovered from superovulated mosaic founder animals carrying CRISPR/Cas9 induced frameshift mutations in *DMD* exon 5 [in the original report of the mosaic founders the mutations were erroneously described as exon 4 mutations (ref. 29 in ref. ^1^)]. In the collective of ICSI-derived DMD rhesus macaques, 4 different *DMD* exon 5 mutations were detected, resulting in the absence of dystrophin and reduced levels of the syntrophin family of adapter proteins associated with the dystrophin protein complex in skeletal muscles. Biopsies taken from limb and back muscles of 6- and 12-month-old animals revealed characteristic signs of muscular dystrophy, including a progressively increased variation in muscle fiber cross-sectional areas and increased proportion of fiber profiles with a central nucleus, as well as progressive fibrosis. These changes were associated with 30- to 100-fold increased serum creatine kinase levels, a marker for muscle damage. Compared to age-matched wild-type (WT) controls, the 1-year-old DMD monkeys displayed several signs of muscle weakness, resembling early-onset muscle weakness observed in DMD patients. Notably, no changes in heart structure and function were detected by echocardiography up to the age of 1 year.

The cellular pathophysiology of DMD in the new rhesus macaque model was further analyzed by single-cell RNA sequencing (scRNAseq) of limb muscle samples from 1-year-old animals. Nine distinct cell clusters were identified, all present in both wild-type (WT) and DMD monkeys. Notably, DMD muscle tissue exhibited a marked increase in immune cells, a reduction in fibro-adipogenic progenitor (FAP) cells, and an elevated number of muscle stem cells (MuSCs).

Sub-clustering of immune cells highlighted a significantly inflamed landscape, with increased pro- and anti-inflammatory macrophage (Mph) subpopulations and cycling Mph, suggesting a highly active inflammatory environment. The presence of regulatory T cells indicated that anti-inflammatory responses were also engaged to counteract chronic muscle injury. Dendritic cells expressing *CLEC9A*, unique to DMD samples, pointed to abnormal muscle damage and impaired regeneration, while the absence of intermediate-stage T cells and elevation in cycling T cells suggested an accelerated T cell maturation process driven by persistent inflammation. The authors interpret their findings as hint for a hyper-activated inflammatory muscle state in DMD macaques, which initially is actively balanced, but later on gives way to pro-inflammatory cells, closely mirroring inflammatory patterns observed in human patients. Whether these are merely responding to chronic muscle decay or initiating additional muscle damage, has not been further elucidated.

Distinct FAP subgroups showed substantial shifts in differentiation under pathological conditions. RNA velocity analysis revealed that FAP subpopulations in DMD macaques differentiated from *CXCL14*^-^*DPP4*^+^ cells towards FAP progenitors, instead of the other way around. Moreover, additional adipogenic differentiation was observed in subpopulations, likely contributing to the fatty infiltration seen in DMD muscles. Importantly, FAPs showed fibrosis independent of the TGF-β pathway, traditionally associated with fibrosis, suggesting alternative pathways may drive fibrotic progression in DMD and potentially presenting new therapeutic targets.

Although MuSC activation was higher in DMD, subpopulation analysis revealed a shift towards differentiation and cycling states, with many cells succumbing to apoptosis or senescence rather than successful regeneration. Similar defects were also seen in human DMD iPSC-derived skeletal myoblasts.^5^ This impaired MuSC function, alongside upregulated fibrosis-related genes, suggests an intrinsic defect in these cells that restricts muscle regeneration and exacerbates disease progression.

A major advantage of the rhesus macaque model is the closer phylogenetic relationship to humans, sharing 93% of the genome sequence, which is notably higher than that of mice, dogs, and pigs. This similarity makes the model especially valuable for studying molecular and cellular disease mechanisms, as highlighted in the study of Ren and colleagues.^1^ The mutations induced in *DMD* exon 5 are within a known mutation hotspot in the *DMD* gene, coding for the actin-binding domain of dystrophin. Notably, mutations affecting *DMD* exon 5 disrupt the production of the full-length 427 kDa dystrophin (Dp427), while shorter isoforms, such as Dp71 in myoblasts, continue to be expressed. Since approximately 10% of patients lack expression of all dystrophin isoforms, the new rhesus macaque model may not accurately represent the phenotype of those individuals.

Despite the significant advantages of a nonhuman primate model, there are important considerations when using rhesus macaques for DMD research. The authors employed advanced assisted reproduction techniques to generate these DMD macaques, which face inherent challenges due to the reproductive characteristics of the species; rhesus macaques reach sexual maturity at 2.5-3.5 years and typically produce only one offspring per pregnancy, limiting scalability for broader studies. Physiological and anatomical aspects further impact the model’s applicability: with an average weight of 1.5–2 kg at one year, the skeletal muscles in rhesus macaques experience significantly less mechanical load than in humans or larger animal models, such as pigs. This is true also for the heart, where anatomical and functional differences may affect the investigation of heart function and stress responses as well as DMD-related cardiac treatments, which is crucial given the progressive heart complications associated with human DMD.^2^ It will be interesting to see if and when dystrophic cardiomyopathy will develop in the new rhesus macaque model for DMD.

In conclusion, the new rhesus macaque model for DMD is a major achievement, complementing the existing animal models and offering unique opportunities for studying the cellular pathophysiology of DMD. It provides researchers with valuable options to better align model selection with specific research objectives in DMD studies.
